# Dose-Dependent Effects on Sphingoid Bases and Cytokines in Chickens Fed Diets Prepared with Fusarium Verticillioides Culture Material Containing Fumonisins

**DOI:** 10.3390/toxins7041253

**Published:** 2015-04-13

**Authors:** Bertrand Grenier, Heidi E. Schwartz-Zimmermann, Sylvia Caha, Wulf Dieter Moll, Gerd Schatzmayr, Todd J. Applegate

**Affiliations:** 1 Department of Animal Sciences Purdue University, West Lafayette, IN 47907, USA; E-Mail: bertrand.grenier@biomin.net; 2 Biomin Research Center, Tulln 3430, Austria; E-Mails: dieter.moll@biomin.net (W.D.M.); gerd.schatzmayr@biomin.net (G.S.); 3 Christian Doppler Laboratory for Mycotoxin Metabolism and Center for Analytical Chemistry, Department for Agrobiotechnology (IFA-Tulln), University of Natural Resources and Life Sciences Vienna, Tulln 3430, Austria; E-Mails: heidi.schwartz@boku.ac.at (H.E.S.-Z.); sylvia.caha@boku.ac.at (S.C.)

**Keywords:** mycotoxins, fumonisins, sphinganine, sphingosine, intestine, mucosal immunity

## Abstract

In chickens, the effect of mycotoxins, especially fumonisins (FB), in the gastrointestinal tract (GIT) is not well documented. Thus, this study in broiler chicks determined the effects of consuming diets prepared with *Fusarium verticillioides* culture material containing FB on intestinal gene expression and on the sphinganine (Sa)/sphingosine (So) ratio (Sa/So; a biomarker of FB effect due to disruption of sphingolipid metabolism). Male broilers were assigned to 6 diets (6 cages/diet; 6 birds/cage) from hatch to 20 days containing 0.4, 5.6, 11.3, 17.5, 47.8, or 104.8 mg FB/kg diet. Exposure to FB altered the Sa/So ratio in all tissues analyzed, albeit to varying extents. Linear dose-responses were observed in the kidney, jejunum and cecum. The liver and the ileum were very sensitive and data fit a cubic and quadratic polynomial model, respectively. Gene expression in the small intestine revealed low but significant upregulations of cytokines involved in the pro-inflammatory, Th1/Th17 and Treg responses, especially at 10 days of age. Interestingly, the cecal tonsils exhibited a biphasic response. Unlike the sphingolipid analysis, the effects seen on gene expression were not dose dependent, even showing more effects when birds were exposed to 11.3 mg FB/kg. In conclusion, this is the first report on the disruption of the sphingolipid metabolism by FB in the GIT of poultry. Further studies are needed to reach conclusions on the biological meaning of the immunomodulation observed in the GIT, but the susceptibility of chickens to intestinal pathogens when exposed to FB, at doses lower than those that would cause overt clinical symptoms, should be addressed.

## 1. Introduction

Mycotoxins are toxic secondary metabolites produced by molds under favorable conditions, and they cause losses for farmers, reduce the value of contaminated feed and affect both animal health and productivity. The major problem associated with animal feed contaminated with mycotoxins is not acute disease episodes, but rather the ingestion of a low level of toxins which may cause an array of metabolic, physiologic, and immunologic disturbances [1].

Among mycotoxins, fumonisins (FB) are metabolites produced by *Fusarium verticillioides* and other fungi, and are common fungal contaminants of corn and other grains. Recent surveys revealed that as much as 55%–65% of finished feed and corn are contaminated worldwide with variable amounts of FB [2,3]. Although the average concentration of positives for FB in commodities ranges from 1 to 3 mg/kg, some feed and feedstuffs reach levels of contamination up to 77 mg/kg. Given the high percentage of corn in poultry rations, substantial amount of FB may be ingested by poultry species. Additionally, it has to be taken into account that FB is concentrated in by-products, such as dried distillers grains with solubles (DDGS) that often serve as animal feed [4]. Overall, chickens seem relatively resistant to mycotoxins in comparison to other farm animals. This natural tolerance has been attributed to a very low intestinal absorption of mycotoxins, a fast transit time of digesta and/or an efficient intestinal metabolism. Nonetheless, very little is known about the effects of mycotoxins on the gastrointestinal tract (GIT) of birds. The poor absorption of FB implies that a substantial non-absorbed portion remains within the lumen of the GIT, exposing the epithelium to high concentrations of toxins [5]. Besides, entero-hepatic recycling may greatly contribute to repeated exposures of the GIT to FB.

Fumonisins are structurally similar to sphingoid bases, sphinganine (Sa) and sphingosine (So), and have been identified as potent inhibitors of sphinganine *N*-acyl transferase (ceramide synthase) [6]. Although FB_2_ and FB_3_ are simultaneously produced with FB_1_, FB_1_ remains the most prevalent (about 70%–80% of the total FB content) and toxic of the FB subspecies. Toxicity and carcinogenicity of FB_1_ are related to the disruption of sphingolipid metabolism that occurs as a result of inhibition of ceramide synthase. This leads to a rapid accumulation of Sa and to a lesser extent of So in biological fluids and tissues, and the Sa/So ratio is well-known to be a dose-dependent early marker of effect to FB. There is increasing evidence for the involvement of sphingolipids in regulating various cellular functions, such as cell-cell interactions, cellular protein and receptor functions, membrane transport, and signal transduction [7]. To the best of our knowledge, the analysis of free sphingoid bases has never been done in samples from the intestine of poultry species. As a matter of fact, this issue in the intestine has only been addressed in three separate studies regardless of the species [8,9,10]. Nonetheless, in the experiments conducted by Enongene *et al.* [8,9], mice were given a single dose of FB_1_ (either oral administration or subcutaneous injection) and epithelial cells from the intestine were collected. Similarly, the data of FB on the immune system are very scarce, particularly at concentrations that do not affect bird performance. It is even noteworthy that no publications have paid attention to the effects of FB on the intestinal immune response of poultry.

The present research aimed to elucidate a plausible dose-response relationship when chickens were fed increasing concentrations of FB, ranging from 5 to 105 mg FB/kg of feed. Analyses of the sphingoid base content and gene expression related to immunity were both carried out to investigate this dose effect. A separate analysis of Sa and So in non-intestinal and intestinal tissues was also done to draw conclusions on tissue sensitivity with regard to the biomarker of effect.

## 2. Results

### 2.1. Effect of FB on the Accumulation of Free Sphingoid Bases and the Sa/So Ratio: Sensitivity of Tissues and Dose-Response Effect

The concentrations of sphinganine (Sa) and sphingosine (So) were evaluated at 10 and 20 days of age in each tissue collected, and the Sa/So ratio was established (Table 1).

**Figure 1:**
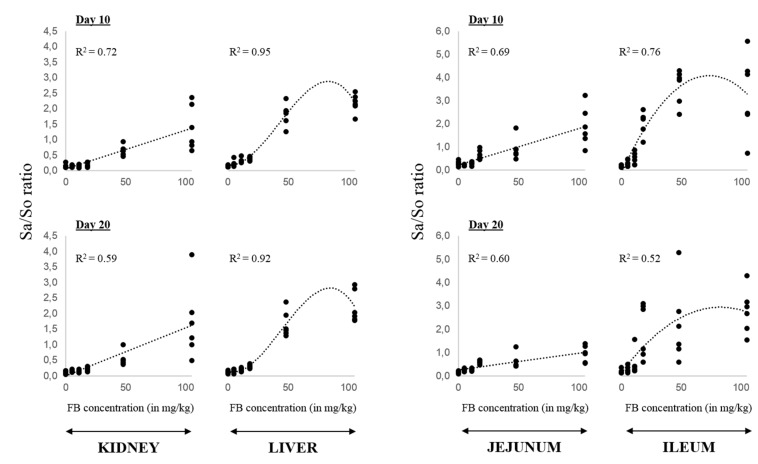
Dose-response effect of FB on the Sa/So ratio in intestinal and non-intestinal tissues. A model of regression was used and the coefficients of determination are indicated. Data in the kidney and jejunum fit linear regression at both sampling times. In the liver, cubic polynomial regressions were found. Data in the ileum follow a quadratic polynomial model. Individual values are displayed, six animals per experimental diet.

Exposure to FB altered the sphingoid base content and the Sa/So ratio in all tissues analyzed, albeit to varying extent. As expected, the liver showed a high sensitivity to FB with 11.3 mg FB/kg able to significantly increase the Sa/So ratio at day 10. This is attributed to a significant elevation of Sa concentration (supplemental data, Table S1). At both days 10 and 20, a cubic polynomial regression (*p* ≤ 0.001) fit to the data set with a marked increase of the Sa/So ratio over 17.5 mg FB/kg (Figure 1). The kidney showed less sensitivity than the liver. Significant effects on the Sa/So ratios were only seen after ingestion of 47.8 mg FB/kg. A linear dose-response was found in that organ (*p* ≤ 0.001). The same linear curve was obtained in the jejunum at both sampling times (*p* ≤ 0.001) and a concentration of 11.3 mg FB/kg was able to significantly affect the Sa/So ratio at day 20 whereas at day 10 significant changes were only observed when birds were fed 17.5 mg FB/kg (Table 1). An unexpected finding was the strong effects seen in the ileum, especially the marked increase in the ratios occurring between 11.3 and 17.5 mg FB/kg (Table 1, Figure 1). Similarly to the liver, this is primarily attributed to an increase of Sa concentrations. Nonetheless, the So content was also significantly increased at day 10 when birds ingested 11.3 mg FB/kg or more (supplemental data, Table S1). Unlike the jejunum, the data in the ileum seemed to follow a quadratic polynomial model (*p* = 0.003 and *p* = 0.031 at days 10 and 20, respectively). We also analyzed the content of Sa and So in the cecum and although the elevation of the ratios was less pronounced than in any other tissues (Table 1), significant differences were observed from 17.5 mg FB/kg and the data fit a linear model (*p* ≤ 0.001).

The analysis also pointed out that the accumulation of sphingoid bases still occurred at 20 days of age, with no obvious difference compared to 10 days of age (except for the cecum, *p* = 0.046).

**Table 1:** Overview of the Sa/So ratios in both intestinal and non-intestinal tissues.

Sa/So ratios	Liver		Kidney		Jejunum		Ileum		Cecum	
FB in Diet (mg/kg)	d10	d20	d10	d20	d10	d20	d10	d20	d10	d20
**0.4 mg/kg**	0.14 ± 0.01	0.12 ± 0.02	0.15 ± 0.02	0.13 ± 0.02	0.31 ± 0.04	0.17 ± 0.02	0.18 ± 0.02	0.19 ± 0.04	0.18 ± 0.04	0.21 ± 0.04
**5.6 mg/kg**	0.23 ± 0.04	0.17 ± 0.02	0.14 ± 0.01	0.16 ± 0.02	0.22 ± 0.01	0.25 ± 0.02	0.31 ± 0.06	0.27 ± 0.05	0.14 ± 0.02	0.19 ± 0.04
**11.3 mg/kg**	0.33 ± 0.03 **	0.19 ± 0.02	0.14 ± 0.02	0.14 ± 0.02	0.29 ± 0.03	0.28 ± 0.02 *	0.51 ± 0.11	0.52 ± 0.21	0.23 ± 0.01	0.13 ± 0.01 *
**17.5 mg/kg**	0.40 ± 0.02 ***	0.31 ± 0.02 **	0.20 ± 0.03	0.21 ± 0.03	0.67 ± 0.08 *	0.60 ± 0.03 ***	2.06 ± 0.20 **	1.93 ± 0.47 *	0.42 ± 0.03 **	0.35 ± 0.03 **
**47.8 mg/kg**	1.82 ± 0.15 **	1.66 ± 0.17 **	0.66 ± 0.07 **	0.55 ± 0.09 *	0.93 ± 0.19	1.12 ± 0.49	3.61 ± 0.30 **	2.37 ± 0.80	0.75 ± 0.18	0.38 ± 0.02 **
**104.8 mg/kg**	2.18 ± 0.12 ***	2.22 ± 0.21 **	1.39 ± 0.29 *	1.72 ± 0.48	1.89 ± 0.34 *	0.95 ± 0.14 *	4.08 ± 1.00 *	2.88 ± 0.48 *	0.62 ± 0.08 *	0.44 ± 0.04 ***
**Probability of diet effect**	<0.001	<0.001	<0.001	<0.001	<0.001	0.011	<0.001	<0.001	<0.001	<0.001

Values are mean ± SEM for six animals. * *p* ≤ 0.05; ** *p* ≤ 0.01; *** *p* ≤ 0.001 compared to control group.

### 2.2. Effect of FB on the Intestinal Immune Defense: Gene Expression in Local and Specific Tissues and Dose-Response Effect

**Figure 2:**
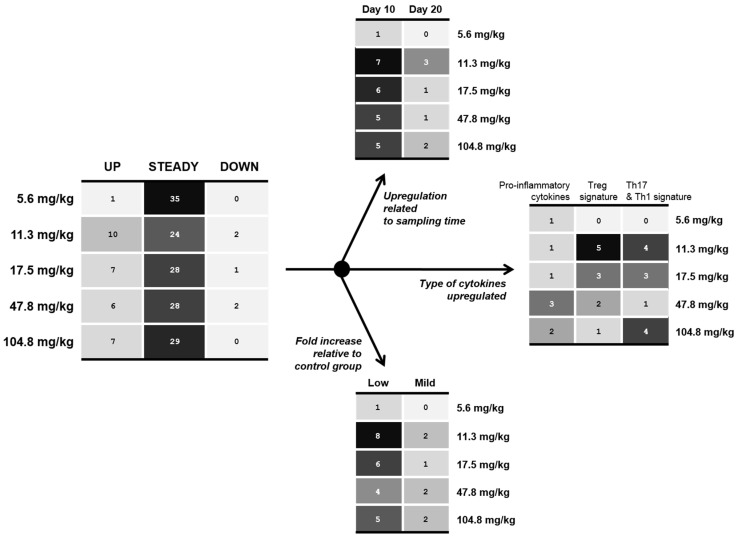
Immunomodulation induced by increasing concentrations of FB on cytokine expression in both the jejunum and ileum—Heat map representation. R software (www.r-project.org) was used to establish the heat maps. This graphical representation reports individual values (here individual cytokines) contained in a matrix that are represented as gray intensity. The heat map on the left refers to all the data in both the jejunum and ileum, and at 10 and 20 days of age. For instance, the value of “10” for 11.3 mg/kg means that 10 significant upregulations of cytokines were found in both jejunum and ileum at days 10 & 20 following ingestion of 11.3 mg FB/kg. This latter heat map was then divided into three sub-heat maps (on the right) analyzing (i) the type of cytokines found to be upregulated, (ii) the effect of sampling time on the upregulation, and (iii) the fold increase of cytokine expression relative to the control group (low, 1.5–2.5 fold; mild, 2.5–4 fold).

**Figure 3:**
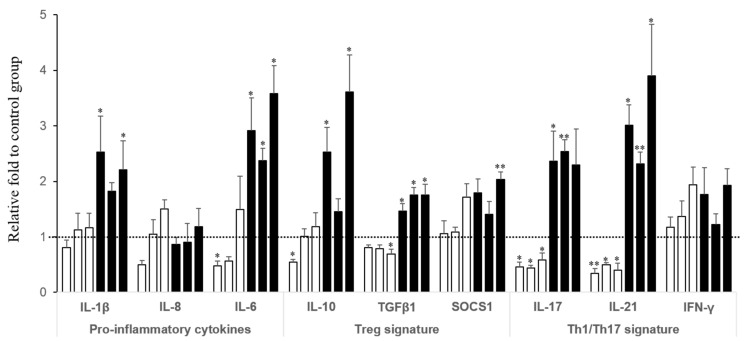
Overview of the biphasic response on cytokine expression in cecal tonsils (for diets containing 11.3, 17.5 and 47.8 mg FB/kg). Expression of cytokines related to inflammation, Treg response, and Th17 & Th1 responses at days 10 and 20. For each cytokine, the first three bars in white (□) correspond to 11.3, 17.5 and 47.8 mg FB/kg at day 10, and the following three bars in black (■) corresponds to 11.3, 17.5 and 47.8 mg FB/kg at day 20. The same order was used for each cytokine. The concentrations of 5.6 and 104.8 mg/kg had induced slight effects and were therefore omitted. Values are mean ± SEM for six animals. * *p* ≤ 0.05; ** *p* ≤ 0.01 compared to control group.

The mRNA level of nine proteins related to immunity (mostly cytokines) was evaluated at 10 and 20 days of age in the small intestine (Table 2), namely the mid-jejunum and the mid-ileum, and in the cecal tonsils, belonging to the gut-associated lymphoid tissue. In contrast to the results of sphingolipids, no linear relationship was observed for any of the immune factors. However, ingestion of FB did significantly modulate the gene expression along the GIT (Table 2, Figure 2 and Figure 3). Based on Table 2, we established heat maps to exemplify our findings in the small intestine (Figure 2). In Figure 2, the main heat map refers to the type of modulation observed in the small intestine (jejunum and ileum).

**Table 2:** Gene expression in the small intestine of chickens fed increasing concentrations of FB—Relative fold to the control group.

FB in diet (mg/kg)	Pro-inflammatory cytokines			Treg signature			Th1 & Th17 signature		
	IL-1β	IL-8	IL-6	IL-10	TGFβ1	SOCS1	IL-17	IL-21	IFN-γ
*Jejunum d10*									
5.6	2.11 ± 0.35 **	1.02 ± 0.14	1.15 ± 0.25	1.40 ± 0.30	1.12 ± 0.14	1.20 ± 0.11	1.20 ± 0.31	1.53 ± 0.30	1.29 ± 0.16
11.3	2.39 ± 0.26 ***	1.74 ± 0.40	0.95 ± 0.13	3.76 ± 0.65 **	0.94 ± 0.07	1.29 ± 0.13	2.32 ± 0.22 *	1.69 ± 0.20	1.61 ± 0.15
17.5	1.96 ± 0.14 **	1.29 ± 0.23	1.52 ± 0.14	3.45 ± 0.82 *	1.12 ± 0.11	1.32 ± 0.19	1.58 ± 0.50	2.40 ± 0.63 *	2.17 ± 0.48 **
47.8	2.08 ± 0.20 **	1.63 ± 0.18	1.82 ± 0.36	2.07 ± 0.59	0.98 ± 0.10	1.39 ± 0.31	1.09 ± 0.17	1.78 ± 0.19	1.61 ± 0.24
104.8	1.80 ± 0.16 *	2.54 ± 0.36 ***	2.37 ± 0.96	2.40 ± 0.61	1.06 ± 0.12	1.31 ± 0.11	0.82 ± 0.09	2.19 ± 0.33 *	1.60 ± 0.25
Probability of the diet effect	0.004	0.004	0.199	0.014	0.765	0.677	0.021	0.101	0.116
*Jejunum d20*									
5.6	1.00 ± 0.30	0.94 ± 0.39	0.88 ± 0.10	0.50 ± 0.14	1.09 ± 0.13	0.90 ± 0.18	1.56 ± 0.77	1.10 ± 0.29	0.65 ± 0.13
11.3	1.66 ± 0.47	2.20 ± 0.81	1.85 ± 0.42	0.86 ± 0.09	1.54 ± 0.12 *	2.03 ± 0.50 *	4.47 ± 1.36 *	1.52 ± 0.20	1.51 ± 0.09
17.5	0.69 ± 0.09	1.17 ± 0.32	0.70 ± 0.09	0.82 ± 0.27	1.83 ± 0.20 **	1.18 ± 0.28	0.59 ± 0.11	1.27 ± 0.33	1.23 ± 0.27
47.8	0.55 ± 0.05 *	1.47 ± 0.71	2.24 ± 1.07	0.84 ± 0.43	1.17 ± 0.09	0.89 ± 0.14	0.77 ± 0.23	1.49 ± 0.73	1.11 ± 0.48
104.8	1.13 ± 0.15	2.14 ± 0.61	1.13 ± 0.24	0.57 ± 0.08	1.73 ± 0.18 **	1.60 ± 0.22	3.41 ± 1.57	1.26 ± 0.25	1.04 ± 0.09
Probability of the diet effect	0.069	0.470	0.258	0.792	0.003	0.052	0.050	0.919	0.269
*Ileum d10*									
5.6	1.55 ± 0.27	1.81 ± 0.57	0.59 ± 0.09	1.25 ± 0.20	1.08 ± 0.11	1.67 ± 0.24	1.72 ± 0.41	1.32 ± 0.19	1.90 ± 0.46
11.3	1.51 ± 0.08	1.89 ± 0.23	0.16 ± 0.04 *	1.32 ± 0.30	1.63 ± 0.15 *	2.13 ± 0.12 *	2.07 ± 0.25 *	1.00 ± 0.17	1.85 ± 0.19 *
17.5	1.49 ± 0.27	2.58 ± 0.50	0.18 ± 0.03 *	1.16 ± 0.21	1.43 ± 0.21	2.18 ± 0.22 *	1.18 ± 0.31	0.56 ± 0.13	1.84 ± 0.16 *
47.8	1.85 ± 0.20 *	2.70 ± 0.39 *	0.27 ± 0.04 *	1.10 ± 0.23	1.27 ± 0.09	2.33 ± 0.49 *	0.96 ± 0.16	0.66 ± 0.17	2.24 ± 0.22 *
104.8	1.48 ± 0.33	1.45 ± 0.21	0.54 ± 0.07	1.19 ± 0.26	1.55 ± 0.42	1.94 ± 0.50	0.92 ± 0.22	1.65 ± 0.14 *	2.11 ± 0.55 *
Probability of the diet effect	0.241	0.046	<0.001	0.947	0.331	0.093	0.035	0.008	0.221
*Ileum d20*									
5.6	0.85 ± 0.18	0.76 ± 0.23	0.82 ± 0.13	0.58 ± 0.14	0.94 ± 0.10	0.67 ± 0.11	1.78 ± 0.45	0.76 ± 0.16	0.80 ± 0.18
11.3	0.74 ± 0.11	0.41 ± 0.08 *	0.81 ± 0.06	0.61 ± 0.15	1.17 ± 0.13	0.70 ± 0.06	1.46 ± 0.23	0.92 ± 0.23	0.75 ± 0.15
17.5	0.79 ± 0.10	0.49 ± 0.19	0.90 ± 0.10	1.27 ± 0.34	1.11 ± 0.24	0.81 ± 0.15	1.62 ± 0.47	0.81 ± 0.14	1.23 ± 0.31
47.8	1.35 ± 0.34	0.66 ± 0.19	0.84 ± 0.07	2.85 ± 1.24 *	1.40 ± 0.13	1.22 ± 0.29	2.09 ± 0.69	2.11 ± 0.36	1.38 ± 0.29
104.8	1.40 ± 0.26	1.53 ± 0.37	0.74 ± 0.09	2.19 ± 0.37	0.93 ± 0.15	1.10 ± 0.15	3.69 ± 0.74 **	1.14 ± 0.11	1.53 ± 0.38
Probability of the diet effect	0.135	0.026	0.772	0.091	0.300	0.215	0.025	0.001	0.257

In gray = significant upregulation compared to control group; Values are mean ± SEM for six animals. * *p* ≤ 0.05; ** *p* ≤ 0.01; *** *p* ≤ 0.001 compared to control group.

Although some genes were found to be modulated following ingestion of FB, the main heat-map also points out that most of the analyzed genes did not show significant differences with the control group. An unexpected finding was that birds exposed to FB at 11.3 mg/kg were the most affected in comparison to other diets (Figure 2). The section “up” of the main heat map was split into three sub-heat maps and analyzed for the effect of sampling time, type of cytokine, and the relative fold increase (Figure 2). The sub-analysis revealed that all types of cytokines were increased, but at 11.3 mg FB/kg the cytokines belonging to the Treg, Th17 and Th1 responses dominated (such as IL-17 and SOCS1). Although the ingestion of 11.3 mg FB/kg induced more upregulation, the relative fold increases in comparison to control group are within the same range (1.5–2.5 fold) for all the diets given to birds. And finally, most of these changes occurred at 10 days of age. Additionally, the jejunum was affected as much as the ileum, unlike the sphingolipid results.

Cecal tonsils (CT) are part of lymphoid tissues in birds that provide specific protection in response to pathogens. In the present study, CT from birds fed contaminated diets with FB exhibited a biphasic response over time (Figure 3). At 10 days of age, CT from chickens showed a significant downregulation of certain cytokines, including IL-6, IL-17 and IL-21 (probability of diet effect: *p* = 0.017, 0.006 and 0.006, respectively). Conversely, those same cytokines and others were found to increase at 20 days of age (probability of diet effect: *p* ≤ 0.001, 0.017, 0.005, 0.029 for IL-6, IL-10, TGFβ1, IL-21, respectively).

### 2.3. Correlation of Free Sphingosine and Immune Factors

**Figure 4:**
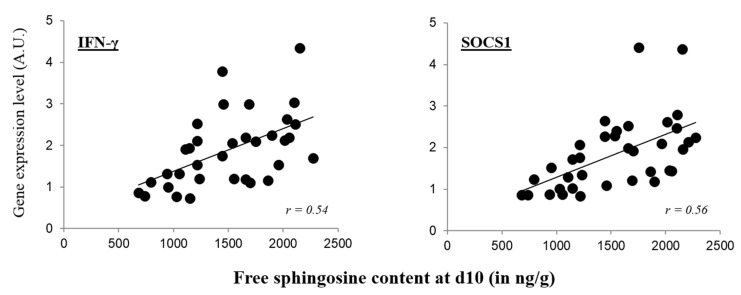
Correlation in the ileum of free sphingosine content and gene expression, at day 10. Individual data (*n* = 36, 6 birds/diets) from the gene expression analysis for IFN-γ and SOCS1 correlated to the sphingosine analysis in the ileum of birds fed the experimental diets. *r* = Pearson correlation coefficient.

As previously mentioned, there is growing evidence for the involvement of sphingolipids in regulating various cellular functions. It was thus of interest to determine a plausible correlation of Sa and So concentrations with our results on gene expression. There was no correlation with Sa and the cytokine expression in both the jejunum and the ileum. Similarly in the jejunum, the increase observed in gene expression was not correlated with the concentrations of So. However, in the ileum at day 10, IFN-γ and SOCS-1 exhibited a positive correlation with the So content (Pearson correlation coefficients of 0.54 and 0.56, for IFN-γ and SOCS1 respectively, both *p* ≤ 0.01; Figure 4).

## 3. Discussion

Poultry are relatively resistant to FB toxicity. Most studies, including the present study (data not shown), did not report any effects on chicken performance up to 80–100 mg FB/kg of feed for 21 days or more [11,12]. As a point of comparison, swine develops lethal pulmonary edema within 4–7 days when fed FB containing feed or culture material at concentrations of ≥ 92 mg/kg [13]. Like mammals, poultry species also exhibit differential sensitivity, with lower concentrations of FB being more detrimental to turkeys and ducks than to chickens [14,15].

Although toxicity differs among animal species, they are all very sensitive to the major cellular effect of FB resulting in the tissue accumulation of sphingoid bases, sphinganine (Sa) and sphingosine (So). This has been extensively studied and attributed to the inhibition of ceramide synthase by FB [16,17]. As expected, increasing concentrations of FB in the diets resulted in a dose-dependent response with elevation of the Sa/So ratios in both intestinal and non-intestinal tissues. This was a direct consequence of FB-induced ceramide synthase inhibition, and not influenced by nutritional factors that can modulate the concentration of So in the GIT. Although the regression analysis indicated linear dose-responses in all the tissues, the data in the liver and ileum fit more a cubic and quadratic polynomial model, respectively, mostly attributed to the ingestion of concentrations over 17.5 mg FB/kg that do not lead to ratios predicted by the linear model. Sa and So are often diverted to sphinganine 1-phosphate (Sa-1-P) and sphingosine 1-phosphate (So-1-P), respectively, which impart cell survival properties [7] whereas the accumulated free sphingoid bases are generally apoptotic [18,19]. This balance might be compromised at high doses of FB and as a result the toxicity would be lethal for the cells. This study point outs that the liver and the ileum are both very susceptible to FB exposure. This effect on the liver was already observed with a concentration as low as 2 mg FB_1_/kg in ducks [20]. However, to our knowledge, this is the first report showing that the sphingolipid metabolism in the intestinal tract of poultry was affected by FB. Although the jejunum of chickens fed the toxin also exhibited significant changes in the Sa/So ratio, the stronger effect in the ileum was unexpected, especially in comparison to the liver. This suggests that the ileal portion was more exposed to FB than any other parts. It has been shown that FB_1_ could interact with cholesterol and/or bile salts while undergoing enterohepatic circulation [21]. Bile salts are reabsorbed actively along the ileum, and therefore, they may expose this intestinal part to significant amount of FB_1_ to facilitate its intestinal absorption. A noteworthy finding is that accumulation of sphingoid bases in the liver was nearly similar to what has been detected in the small intestine. The very poor intestinal absorption of FB (less than 5% in different species, review in [5,22]) implies that the GIT should be more sensitive to the effect of FB, at least in terms of sphingolipid disruption. Although many reasons may account for this discrepancy (differential activity/expression of enzymes involved in sphingolipid metabolism, base degradation and phosphorylation), it is also tempting to ascribe this effect to the life span of hepatic and intestinal cells. Indeed, cell life time can vary considerably by tissue, ranging from 2–6 days for intestinal epithelial cells to 6–18 months for cells in the liver. This suggests a faster elimination of free sphingoid bases in the intestine than in the liver.

An unexpected finding was also the lack of dose-response observed in the analysis of gene expression in the GIT. Increasing concentrations of FB in the diets did not result in an increasing effect on mucosal immunity. Although the changes observed in cytokine expression were low and transient in the small intestine (about 2 fold-increase and mostly at day 10), the analysis revealed that the total effects at 11.3 mg FB/kg were more significant than for any other FB concentrations. Very recently, Minervini *et al.* [23] reported that the toxic effects induced by FB_1_ in the human intestinal cell line, such as on lipid peroxidation, were not dose-dependent. Nonlinear relationships, often referred to as non-monotonic and low dose responses in toxicology, have been commonly reported in studies of natural hormones and endocrine-disrupting chemicals, such as bisphenol A [24]. This major concept has challenged the traditional dogma of “the dose makes the poison,” because some compounds can have effects at low doses that are not predicted by effects at higher doses. Somewhat similar results have been reported for another mycotoxin, aflatoxin that follow a pattern of hormesis, characterized by low-dose stimulation and high-dose inhibition [25,26]. A close correlation between FB-induced disruption of sphingolipid metabolism and the onset of toxicity *in vivo* has been often reported, especially in rodents [27,28]. In mice, Riley *et al.* [27] clearly showed a correlation between free Sa in the liver and histopathological findings as well as with hepatic enzyme concentration. In the present study, it seems that Sa did not contribute, or did so only slightly, to the effects seen on cytokine expression in the small intestine as no dose-response or correlation was observed. On the other hand, the So analysis revealed that ileal So content at day 10 was similar for diets with 11.3, 47.8 and 104.8 mg FB/kg (1822 ± 179, 1823 ± 84, 1818 ± 159 ng/g, respectively) and significantly different from the control group (919 ± 73 ng/g). A similar trend in So accumulation has been reported in the study of He *et al.* [29] using increasing concentrations of FB_1_ in mice. In addition, a positive correlation in the ileum was found with SOCS1 and IFN-γ (Figure 4). So and particularly its respective phosphate So-1-P are important lipid mediators implicated in many biological processes, such as inflammation and cancer, regulation of lymphocyte trafficking and differentiation, and cytokine and chemokine production [30,31]. Nonetheless, the So content in the jejunum was not as much affected, and therefore, the contribution of So to our immunological findings seems rather small. The use of culture material containing FB instead of purified FB also suggests that unidentified compounds (e.g., cell wall of fungi, other mycotoxins not analyzed) could have contributed to the effects observed. In line with that, a study reported that bacterial colonization in pigs was higher in pigs treated with a crude extract containing FB_1_ than in the animals treated with the purified toxin [32]. An appropriate control in our study would have been a control diet formulated with an equal amount of *F. verticillioides* culture material from a non-producing strain. Further studies are needed to confirm that the effects seen on gene expression are specifically due to FB exposure.

Until recently, not much was known about the effects of mycotoxins, especially low doses, on the GIT. Nonetheless, recent data, especially using the mycotoxin deoxynivalenol, pointed out some intestinal alterations that could predispose animals to intestinal inflammation and enteric infections [5,33,34,35]. Typical changes observed during chronic intestinal inflammation include alterations of morphology, permeability, motility, nutrient absorption and, microbiota and cytokine balance. Therefore, it is premature at this point to claim that ingestion of FB resulted in low-grade inflammation based only on our immunological findings (and on the very few published studies regarding FB effects on the intestine of chickens). However, the effects on the cytokine balance and the type of T cell involved are discussed. Using intestinal *in vitro* and *ex vivo* approaches, Cano *et al.* [34] showed that deoxynivalenol induced an early intestinal inflammatory response leading to the activation of Th17 cells. This subset of T helper lymphocytes was recently described as an important mediator of mucosal immunity, defense against extracellular pathogens and inflammation [36]. Although our samples were collected at day 10, the increase in mRNA expression encoding for IL-8 and IL-1β might evoke a similar and early general state of inflammation. The inflammation generated at the epithelial level could elicit the activation of the population of Th17 cells by triggering further communication with immune cells of the lamina propria. Th17 cells produce IL-17 and IL-21, and in the small intestine of birds fed FB, especially at 11.3 mg/kg, IL-17 expression was found upregulated. Interestingly, this higher expression of cytokines related to Th1/Th17 cells concur with an increased level of cytokines produced by Treg cells, IL-10 and SOCS1. SOCS1 is highly expressed in Treg cells, and defined as an important mechanism for the negative regulation of the cytokine-JAK-STAT pathway and uncontrolled IFN-γ signaling, such as in inflammatory conditions [37,38]. Among the current findings on cytokine expression, both SOCS1 and IFN-γ were found to be significantly increased in the small intestine, and not only at 11.3 mg FB/kg. Also, these proteins were the only ones to show a close correlation when paired with So content, regardless of the experimental concentrations used (Figure 4). Taken together, these findings might suggest that SOCS1 protects Treg cells from negative effects of inflammatory cytokines which promote the conversion into Th1/Th17-like effector cells, and helps the birds to attenuate cytokine signaling and regulate the cytokine balance. This may account for the results at 20 days of age, given much fewer effects were seen.

An interesting finding in the present study concerns the biphasic response observed in the cecal tonsils (CT) of birds exposed to FB. Unlike the small intestine, FB ingestion seemed to downregulate cytokine expression first, followed by an upregulation when birds became older. That point warrants further investigation, but it is highly plausible that the differential anatomy, cell content, immune functions of the CT and the jejunum/ileum accounts for these findings. CT belong to the gut-associated lymphoid tissue, encompassing mainly immune cells, such as different types of lymphocytes and to lesser extent antigen-presenting cells. This lymphoid tissue provides specific protection through the development of an acquired (adaptive) response, and leads ultimately to the clearance of pathogens. By contrast, the small intestine provides an innate response through the local action of intestinal epithelial cells (major sources of cytokines and chemokines as well), mucous secretion and certain immune cells residing in the lamina propria, such as intraepithelial lymphocytes and natural killers. CT are rudimentary in chicken embryos and at hatch have only low numbers of lymphocytes. A major wave of lymphocyte colonization occurs at 4 d of age and lymphocytes become functionally mature during the first two weeks of life [39]. Functional maturity of the chicken gut is linked to the maturation of the local immune system which seems to be provided during the first week by the innate immune defense [40]. This delayed maturation and colonization of lymphocytes in CT might explain the low expression of cytokines observed at 10 d of age in birds exposed to FB in comparison to the results on jejunum/ileum. FB could have retarded the capabilities of development of functional lymphocytes. Also, a differential sensitivity of cells to FB is possible. As previously mentioned, CT are rich in lymphocytes compared to the small intestine, and these cells might be very sensitive to FB [41,42]. At 20 d of age, this decrease in cytokine expression was no longer observed, and similarly to the small intestine at 10 d of age, most cytokines assessed were increased. It is tempting to speculate that once lymphocytes in CT were mature enough, while delayed by FB, these cells were functionally active and also exhibited a pro-inflammatory response, as depicted by the mRNA levels of IL-1β and IL-6 (Figure 3). Also, it has been recently demonstrated in chicken that CD4^+^CD25^+^ cells have Treg properties (high IL-10, TGFβ amounts), and are present in high numbers in mucosal regions like CT [43,44]. Treg-derived IL-10 showed high expression in the present study and this elevation may act as an anti-inflammatory response to the increase observed in IL-17 and IL-21 expression, two cytokines related to the Th17 response, as previously mentioned.

## 4. Experimental Section

### 4.1. Experimental Birds, Housing, Diet Formulation and Sampling

All animal care and use procedures for the experiment were approved by the Purdue University Animal Care and Use Committee. A 20-day feeding study using a crude culture containing FB was conducted with 1d-old male broilers (Ross 708). The culture material was from *Fusarium verticillioides* (M-3125, [45]), which was grown on rice, homogenised and freeze-dried, and FB concentration was determined (18.37 mg FB_1_ + FB_2_ per g of culture material). Six replicate cages (6 birds per cage) were fed experimental diets containing increasing concentrations of FB lyophilisate and formulated to contain nominal concentrations of 0, 5, 10, 20, 50 and 100 mg/kg of FB. The actual concentrations, as well as the natural presence of other major mycotoxins, were determined analytically (conducted by Quantas and IFA, Tulln, Austria; [46], Table 3), and reported in the text and tables. Diet analyses, including the ingredient formulation and nutrient composition, are reported in Table 3.

Birds were housed in stainless-steel battery brooders equipped with nipple-type waterers and thermostatically controlled heaters. The mortality of birds was recorded daily. Body weight and feed intake were measured on 0, 10 and 20 days of age. All of the birds were euthanized by an overdose of carbon dioxide (36 birds/diet). However, only 6 birds/diet (1 bird/cage, 6 cages/diet) were sampled for analyses. Liver, kidney, jejunum, ileum and cecum were taken at both 10 and 20 d of age, flash frozen in liquid nitrogen and stored at −80 °C until sphingolipid analysis. Similar segments of the small intestine, as well as the cecal tonsils, were taken from the same birds (1 bird/cage, 6 cages/diet), and placed in cryovials containing RNAlater (Ambion Inc., Austin, TX, USA) for subsequent RNA isolation.

**Table 3:** Diet formulation, nutrient specification and mycotoxin content.

Item	Starter Diet					
Ingredient (% of diet)						
Corn	54.18					
Soybean meal (48% CP)	38.05					
Soy oil	3.52					
Sodium chloride	0.47					
DL-Methionine	0.25					
Threonine	0.07					
l-Lysine, HCl	0.10					
Limestone	1.68					
Monocalcium phosphate	1.33					
Vitamin and mineral premix ^1^	0.35					
Nutrient composition (calculated)						
ME, kcal/kg	3066					
CP, %	22.43					
Ca, %	1.01					
Non-phytate phosphorus, %	0.43					
Met, %	0.59					
Thr, %	0.92					
Lys, %	1.34					
Analyzed composition	**Target and actual concentration of mycotoxins in the formulated diets (in mg/kg of feed)**					
	***0***	***5***	***10***	***20***	***50***	***100***
Fumonisin B_1_	0.26	3.9	7.1	11.0	31.7	66.9
Fumonisin B_2_	0.09	1.4	3.4	5.1	12.4	27.5
Fumonisin B_3_	0.02	0.3	0.8	1.4	3.7	10.4
***Total Fumonisins***	***0.37***	***5.6***	***11.3***	***17.5***	***47.8***	***104.8***
Deoxynivalenol	Range from 0.236 to 0.344 mg/kg					
Zearalenone	Range from 0.015 to 0.029 mg/kg					
Aflatoxin B_1_	<LOD; LOD = 0.3 μg/kg					
Ochratoxin A	<LOD; LOD = 0.2 μg/kg					
T-2 toxin	<LOD; LOD = 25 μg/kg					

^1^ Supplied the following per kilogram of diet: Iron, 71.6 mg; copper, 11.0 mg; manganese, 178.7 mg; zinc, 178.7 mg; iodine, 3.0 mg; selenium, 0.4 mg. vitamin A, 18,904 IU; vitamin D3, 9480 IU; vitamin E, 63.0 IU; vitamin K activity, 6.4 mg; thiamine, 3.2 mg; riboflavin, 9.4 mg; pantothenic acid, 34.7 mg; niacin, 126.0 mg; pyridoxine, 4.7 mg; folic acid, 1.6 mg; biotin, 0.5 mg; vitamin B12, 35.4 µg; choline, 956.9 mg. Bold and italic part, please explain.

### 4.2. Experimental Parameter Measures

#### 4.2.1. Sphingolipid Analysis (day 10 & 20)

Tissue samples were homogenized on ice in the four-fold volume of cold potassium dibasic phosphate buffer (50 mM, pH 7). For sphingolipid analysis, 200 µL aliquots of the homogenates were shaken with 0.6 mL of methanol/acetonitrile (50/50, *v*/*v*) at room temperature for 30 min. After centrifugation, the pellets were re-extracted with 0.3 mL of methanol/water (80/20, *v*/*v*), the combined supernatants were evaporated to dryness under compressed air and the residues were taken up in 400 µL of methanol/water (80/20, *v*/*v*) and centrifuged. HPLC-MS/MS analysis was conducted on an Agilent 1290 series UHPLC system coupled to a 4000 QTrap mass spectrometer (AB Sciex, Foster City, CA, USA). Chromatographic separation was achieved on a C18 Gemini column (150 × 4.6 mm, 5 µm, Phenomenex, Aschaffenburg, Germany) at a flow rate of 0.9 mL/min in gradient elution. Mobile phase A consisted of methanol/water/formic acid (40/59.9/0.1, *v*/*v*/*v*), mobile phase B of methanol/formic acid (99.9/0.1, *v*/*v*). Gradient elution started with an isocratic period at 35% B for 0.2 min and continued with a linear increase to 100% B within further 6.8 min. An isocratic hold from 7.0 to 10.4 min was followed by a rapid change back to the starting conditions within 0.1 min. Finally, the column was re-equilibrated from 10.5 to 12.5 min. The injection volume was 1 µL and the LC stream was directed to MS between 3.8 and 9.5 min. Tandem mass spectrometric detection was carried out in positive ion mode after electrospray ionization at 550 °C (ion spray voltage: 4200 V). The declustering potentials (DP), quantifier (quant) and qualifier (qual) transitions were DP 56 V, quant: 300.3-> 282.3 (CE 17 eV), qual: 300.3-> 252.2 (CE 25 eV) for So and DP 71 V, quant: 302.3-> 284.3 (CE 21 eV), qual: 302.3-> 60.1 (CE 41 eV) for Sa. Analyst^®^ software version 1.5.2 (AB Sciex, Foster, CA, USA, 2011) was used for instrument control and data evaluation. Concentrations of So and Sa (both purchased from Avanti Polar Lipids, Inc., Alabaster, Alabama, AL, USA) and the Sa/So ratio in tissue samples were determined on the basis of neat standard calibration functions recorded between 3 and 1000 ng/mL. Recoveries of extraction were determined for Sa and So in the liver and the ileum. All values were ≥70% (70% for Sa and 85% for So in the liver and 80% for Sa and 85% for So in the ileum) and therefore considered acceptable following the EU requirements on methods for *Fusarium* toxins (recovery limits between 70%–120%). According to our investigations, the use of C20 sphingosine as internal standard is problematic since C20 sphingosine occurs naturally in tissues of FB treated animals.

#### 4.2.2. Gene Expression (day 10 & 20)

Tissues were processed in lysing bead tubes containing guanidine-thiocyanate acid phenol (QIAzol reagent, Qiagen, Valencia, CA, USA) for use with the FastPrep-24 (MP Biomedicals, Solon, OH, USA). Concentrations, integrity and quality of RNA were determined spectrophotometrically using Nanodrop ND1000 (Fisher Scientific, St. Louis, MO, USA). Two micrograms of total RNA were treated with DNase I (Sigma Aldrich, St. Louis, MO, USA) as some genes were lacking introns. RNA was reverse-transcribed using M-MLV reverse transcriptase (Promega, Madison, WI, USA). Real-time PCR was performed using iCycler iQ real-time PCR detection system (Bio-Rad, Hercules, CA, USA) with the iQ SYBR Green Supermix (Bio-Rad). Thermal cycling conditions for the PCR reactions were 95 °C for 5 min followed by 40 cycles of 95 °C for 10 s, then 55 °C for 20 s, and finishing at 72 °C for 20 s. RNA non-reverse transcript was used as the non-template control for verification of a no genomic DNA amplification signal. Each sample was assessed in duplicate on two separate plates (in triplicate if high coefficient of variation). Specificity of PCR products was checked at the end of the reaction by analyzing the curve of dissociation. In addition, the size of amplicons was verified by electrophoresis. The sequences of the primers used are detailed in Table 4. Amplification efficiency and initial fluorescence were determined using the DART-PCR method [47]. Then, values obtained were normalized by both housekeeping genes glyceraldehyde 3-phosphate dehydrogenase (GAPDH) and ribosomal protein L4 (RPL4). Finally, gene expression was expressed relative to the control group.

**Table 4:** Nucleotide sequence of primers for real-time PCR.

Gene	Primer sequence	Amplicon		Ensembl access	References
		Size	Intron ^1^		
*Housekeeping genes*					
GAPDH	F (300 nM) TCCTAGGATACACAGAGGACCA R (300 nM) CGGTTGCTATATCCAAACTCA	151 bp	2*(499)*	ENSGALG00000014442	Present study
RPL4	F (300 nM) TTATGCCATCTGTTCTGCC R (300 nM) GCGATTCCTCATCTTACCCT	235 bp	2*(893)*	ENSGALG00000007711	Present study
*Pro-inflammatory cytokines*					
IL-1β	F (300 nM) GCATCAAGGGCTACAAGCTC R (300 nM) CAGGCGGTAGAAGATGAAGC	131 bp	1*(87)*	ENSGALG00000000534	[48]
IL-6	F (300 nM) GAATGTTTTAGTTCGGGCACA R (300 nM) TTCCTAGAAGGAAATGAGAATGC	130 bp	0	ENSGALG00000010915	Present study
IL-8	F (300 nM) GCGGCCCCCACTGCAAGAAT R (300 nM) TCACAGTGGTGCATCAGAATTGAGC	146 bp	2*(1210)*	ENSGALG00000011670	Present study
*Treg signature*					
IL-10	F (300 nM) GCTGAGGGTGAAGTTTGAGG R (300 nM) AGACTGGCAGCCAAAGGTC	121 bp	2*(1127)*	ENSGALG00000000892	Present study
TGFβ1	F (300 nM) CGGGACGGATGAGAAGAAC R (300 nM) CGGCCCACGTAGTAAATGAT	258 bp	no data		[49]
SOCS1	F (300 nM) CAAGCGGATTTCAGTAGCATC R (300 nM) GGCTCAGACTTCAGCTTCTCA	110 bp	no intron	ENSGALG00000007158	Present study
*Th17 & Th1 signature*					
IL-17	F (300 nM) TATCAGCAAACGCTCACTGG R (300 nM) AGTTCACGCACCTGGAATG	110 bp	1*(666)*	ENSGALG00000016678	Present study
IL-21	F (300 nM) GCTTTCAAAGACAATTGACCATC R (300 nM) TACAGCTGTGAGCAGGCATC	106 bp	2*(3765)*	ENSGALG00000011844	Present study
IFN-γ	F (300 nM) AGCTGACGGTGGACCTATTATT R (300 nM) GGCTTTGCGCTGGATTC	259 bp	2*(998)*	ENSGALG00000009903	[49]

^1^ number of introns spanned in the design of primers, the brackets report the total size of introns (in bp). GAPDH, Glyceraldehyde 3-phosphate dehydrogenase; IFN-γ, Interferon-γ; IL, Interleukin; RPL4, Ribosomal protein L4; SOCS1, Suppressor of cytokine signaling 1; TGFβ1, Transforming growth factor β 1.

### 4.3. Statistics

Data were subjected to analysis of variance using IBM SPSS statistics software (Version 19.0, IBM corp., New York, NY, USA, 2010). The data were first analyzed as a completely randomized design with the experimental unit as a cage of birds (1 bird/cage) to examine the overall effects of diets. The Fisher’s Least Significance Difference (LSD) test was then used as a *post hoc* test or the Games-Howell test in case the equal variances were not assumed (Levene statistic). Orthogonal polynomial contrasts were used to determine linear, quadratic and cubic responses to FB levels in feed. The Pearson correlation coefficient was also determined as a measure of the linear correlation between sphingosine content and cytokine expression in the ileum. In addition, the heat maps were established with a separate program, the R software package (www.r-project.org). Statements of significance were *p* ≤ 0.05 unless noted otherwise.

## 5. Conclusions

In conclusion, this study has shown for the first time in poultry that the sphingolipid metabolism is also disrupted in the intestine following ingestion of FB (in a dose-dependent manner), with the ileum being very sensitive. The ileum, along with the jejunum, also exhibited a low but significant modulation of the cytokine balance but not in a dose-dependent manner. This suggests that either chickens might be less sensitive than other species to fluctuations and disturbances in sphingolipid metabolism or the effects seen on cytokine expression were not only related to FB ingestion but also to the presence of unidentified compounds in the culture material containing FB. However, the effects induced by the culture material containing 11.3 mg FB/kg, below the EU and US recommendations (20 mg/kg and 50 mg/kg, respectively, in poultry feed) need further investigation, as do the effects of FB during the very first days of exposure. The study of the interaction of FB with intestinal pathogens could shed some light on the meaning of our immunological findings, and also determine the susceptibility of chicken fed FB to pathogens such as those very recently demonstrated with deoxynivalenol [50]. Finally, this study also highlights that endpoints typically examined at high doses, such as animal performance, are not as sensitive as other measures when studying lower doses.

## Supplementary Materials

Supplementary materials can be accessed at: http://www.mdpi.com/2072-6651/7/4/1253/s1.

## Abbreviations

CT: Cecal Tonsils
FB: Fumonisins
GIT: Gastrointestinal Tract
Sa: Sphinganine
So: Sphingosine
So-1-P: Sphingosine-1-Phosphate
