# Supplementary material for: Dose-Dependent Effects on Sphingoid Bases and Cytokines in Chickens Fed Diets Prepared with Fusarium Verticillioides Culture Material Containing Fumonisins
Source: Toxins (Basel). 2015 Apr 13;7(4):1253–72. doi: 10.3390/toxins7041253 (PMC4417966; doi:10.3390/toxins7041253)
Supplement: Supplementary file 1 [file toxins-07-01253-s001.pdf]

Supplementary Materials

Table S1. Concentrations of sphinganine (Sa) and sphingosine (So) in non-intestinal and intestinal tissues.

**Sphingoid bases**	**Liver**		**Kidney**		**Jejunum**		**Ileum**		**Cecum**	
**Sa (ng/g)**	**d10**	**d20**	**d10**	**d20**	**d10**	**d20**	**d10**	**d20**	**d10**	**d20**
**0.4 mg/kg**	674 ± 119	658 ± 140	172 ± 30	111 ± 15	237 ± 42	210 ± 15	168 ± 16	231 ± 29	722 ± 171	742 ± 222
**5.6 mg/kg**	803 ± 178	542 ± 125	124 ± 18	76 ± 12	244 ± 21	288 ± 32	445 ± 134	268 ± 91	640 ± 154	268 ± 94
**11.3 mg/kg**	1434 ± 202	661 ± 83	157 ± 17	140 ± 34	277 ± 31	323 ± 57	943 ± 254	574 ± 255	744 ± 136	141 ± 29
**17.5 mg/kg**	1890 ± 181 **	1346 ± 108 *	264 ± 50	146 ± 30	638 ± 122	586 ± 97 *	2885 ± 207 ***	2751 ± 638 *	2191 ± 262 *	428 ± 79
**47.8 mg/kg**	10795 ± 2698 *	14530 ± 2042 **	2636 ± 712	1463 ± 346 *	1311 ± 420	1164 ± 477	6640 ± 739 **	5345 ± 1585 *	1378 ± 279	765 ± 164
**104.8 mg/kg**	23503 ± 2572 **	21214 ± 1756 ***	5851 ± 1702	5639 ± 2196 *	2143 ± 476 *	1198 ± 376	7741 ± 2284 *	6862 ± 1139 *	2424 ± 478 *	581 ± 63
Probability of diet effect	$p < 0.001$	$p < 0.001$	$p < 0.001$	$p = 0.001$	$p < 0.001$	$p = 0.022$	$p < 0.001$	$p < 0.001$	$p < 0.001$	$p = 0.006$
**So (ng/g)**										
**0.4 mg/kg**	4620 ± 662	4816 ± 730	1139 ± 151	895 ± 120	750 ± 36	1340 ± 205	919 ± 73	1274 ± 75	4433 ± 1308	2925 ± 596
**5.6 mg/kg**	3390 ± 272	3045 ± 384	847 ± 60	501 ± 86	1121 ± 86 *	1185 ± 160	1349 ± 185	907 ± 124	4734 ± 1265	1396 ± 343
**11.3 mg/kg**	4330 ± 506	3443 ± 280	1196 ± 105	980 ± 171	970 ± 90	1186 ± 213	1822 ± 179 **	1121 ± 90	3492 ± 867	1120 ± 289
**17.5 mg/kg**	4756 ± 390	4381 ± 158	1266 ± 98	750 ± 181	934 ± 84	1010 ± 182	1459 ± 141 *	1481 ± 76	5145 ± 515	1203 ±185
**47.8 mg/kg**	5748 ± 1143	8741 ± 793 *	3979 ± 907	2587 ± 551 **	1299 ± 124 **	1128 ± 121	1823 ± 84 **	2305 ± 296	2565 ± 813	2015 ± 451
**104.8 mg/kg**	10797 ± 1056 **	9698 ± 625 **	3853 ± 485 *	2659 ± 411 **	1109 ± 124 *	1163 ± 237	1818 ± 159 **	2441 ± 237 *	4189 ± 910	1445 ± 95
Probability of diet effect	$p < 0.001$	$p < 0.001$	$p < 0.001$	$p < 0.001$	$p = 0.008$	$p = 0.900$	$p < 0.001$	$p < 0.001$	$p = 0.497$	$p = 0.013$

Values are mean ± SEM for six animals. * $p \leq 0.05$; ** $p \leq 0.01$; *** $p \leq 0.001$ compared to control group.
